# *Bacillus cereus* in Infant Foods: Prevalence Study and Distribution of Enterotoxigenic Virulence Factors in Isfahan Province, Iran

**DOI:** 10.1155/2013/292571

**Published:** 2013-05-27

**Authors:** Ebrahim Rahimi, Fahimeh Abdos, Hassan Momtaz, Zienab Torki Baghbadorani, Mohammad Jalali

**Affiliations:** ^1^Department of Food Hygiene and Public Health, College of Veterinary Medicine, Islamic Azad University, Shahrekord Branch, P.O. Box 166, Shahrekord, Iran; ^2^Department of Food Science and Technology, College of Agriculture, Islamic Azad University, Shahrekord Branch, Shahrekord, Iran; ^3^Department of Microbiology, College of Veterinary Medicine, Islamic Azad University, Shahrekord Branch, Shahrekord, Iran; ^4^Infectious Disease and Tropical Medicine Research Center and School of Food Science and Nutrition, Isfahan University of Medical Sciences, Isfahan, Iran

## Abstract

This study was carried out in order to investigate the presences of *Bacillus cereus* and its enterotoxigenic genes in infant foods in Isfahan, Iran. Overall 200 infant foods with various based were collected and immediately transferred to the laboratory. All samples were culture and the genomic DNA was extracted from colonies with typical characters of *Bacillus cereus*. The presences of enterotoxigenic genes were investigated using the PCR technique. Eighty-four of two hundred samples (42%) were found to be contaminated with *B. cereus* with a ranges of 3 × 10^1^–9.3 × 10^1^ spore per gram sample. Totally, *entFM* had the highest (61.90%) incidences of enterotoxigenic genes while *hblA* had the lowest (13.09%) incidences of enterotoxigenic genes. Overall, 6.7% of *B. cereus* isolates had all studied enetrotoxigenic genes while 25.5% of *B. cereus* strains had all studied enetrotoxigenic genes expectance *bceT* gene. Thisstudyisthe first prevalence report of *B. cereus* and its enterotoxigenic genes in infant foods in Iran. Results showed that the infant food is one of the main sources of enterotoxigenic genes of *B. cereus* in Iran. Therefore, the accurate food inspection causes to reducing outbreak of diseases.

## 1. Introduction

Baby foods are the primary source of nutrition for kids before they are able to digest other types of food. Their high values for proteins, minerals, fats, and vitamins are undeniable. In a day, millions of babies use these foods in the world. Babies have the weak immune system and any infection in their foods causes their illness. Therefore, the hygienic quality of baby foods is very important but sometimes it will be changed and several infections and illness occur. 

Foodborne diseases are a worldwide growing health problem involving a wide spectrum of illnesses caused by bacterial, viral, parasitic, or chemical contamination of food. A previous report of the World Health Organization (WHO) showed that the *Bacillus cereus *(*B. cereus*) is the most common foodborne bacterium in pasteurized food products [1–3]. 

The* B. cereus* is a Gram-positive and rod-shaped bacterium which is responsible for causing diarrhea, emesis, fatal meningitis, and spoilage of different food products [4, 5]. The *B. cereus* is a spore former organism. Therefore, there is a risk in its transmission through processed, pasteurized, sterilized, and heat-treated food products. The spore of this bacterium can survive in low and high temperatures. The most common source of this bacterium is liquid food products, milk powder, mixed food products, and, with particular concerns, the baby formula industry [6, 7]. The occurrence of *B. cereus* as a contaminant of baby food was previously reported [8, 9].

Food spoilage, diarrhea, emesis, and other complications of *B. cereus* are caused by several virulence genes. Virulence genes of *B. cereus* have been ascribed to different extracellular factors. Two of these virulence factors are protein complexes, that is, the hemolysin BL (HBL) [10] and the nonhemolytic enterotoxin (NHE) [11]. Other factors are single-gene products encoded by *entFM* (enterotoxin FM) and *bceT* (*B. cereus* enterotoxin) [12]. 

It is important to know which genes of *B. cereus* are endemic in various regions and samples. Besides, the epidemiology and prevalence of *B. cereus *are indeed unknown in Iranian baby foods. Therefore, the present study was carried out in order to study the prevalence of *B. cereus* and its enterotoxigenic virulence factors in infant foods in Isfahan province, Iran. 

## 2. Materials and Methods

### 2.1. Samples and Identification of *B. cereus*

Overall 200 baby food samples including baby food with rice and milk based (*n* = 50), baby food with wheat and milk based (*n* = 50), baby food with wheat, honey, and milk based (*n* = 50), and baby food with wheat, banana, and milk based (*n* = 50) were purchased from the supermarkets of  Isfahan, Iran. All of these products were pasteurized and after collection were kept under refrigeration in plastic bags; information about dates of production and of assigned shelf lives was not presented. 

First, tenfold 10 g of each sample was added into 90 mL 0.1% (wv^−1^) peptone water. The samples were well mixed and homogenized by vigorous vortexing at room temperature for 3 min. Tenfold dilution was prepared in 20% (vv^−1^) glycerol-peptone water. A 50 *μ*L aliquot from this dilution was inoculated into 5 mL Nutrient Broth (NB) (Applichem) and incubated at 37°C for 18 h with shaking at 150 rpm. The tubes were pasteurized at 80°C for 10 min to eliminate nonsporulating bacteria. The suspension was streaked onto chromogenic *B. cereus* agar (BCA) supplemented with chromogenic *B. cereus* selective supplement (Oxoid). The plates were incubated at 37°C overnight and blue/green colonies were subcultured on chromogenic BCA until obtaining a pure culture. After identification by biochemical tests (Gram staining and catalase test), the isolated strains were stored in sterile NB containing 20% (vv^−1^) glycerol at −80°C. The colonies with the typical characters of *B. cereus* were tested using the polymerase chain reaction (PCR) method [13, 14].

### 2.2. DNA Extraction

Genomic DNA was extracted from the culture positive colonies. Genomic DNA was extracted by freezing first and then boiling the cells. Strains were grown at 37°C for 16 h. A loopful of cells was scraped off from NA plate and resuspended in 150 *μ*L sterile water. After 20 min of freezing at −80°C, the samples were boiled for 10 min to lyse the cells completely. Cell debris was removed by centrifugation (11000 g, 10 s). The supernatant containing genomic DNA was stored at −20°C.

### 2.3. PCR for Detection of Virulence Genes

The strains were tested for the presence of enterotoxin genes. The primer sets used in this study are presented in Table 1. Each amplification process was performed in a 50 *µ*L reaction mixture containing 100 ng of genomic DNA as the template, 5 *µ*L of 10x reaction buffer (100 mMTris-HCl (pH 8.8), 500 mMKCl, 0.8% (vv^−1^) Nonidet P-40, and 1.5 mM MgCl_2_), 10 *µ*M of each of the primers, 0.2 mM of each of the four dNTPs (Fermentas), and 2 U Taq DNA polymerase (Fermentas). Reactions were initiated at 95°C for 5 min, followed by 30 cycles of 95°C for 1 min, 58°C for 1 min, 72°C for 1 min, and a final elongation step at 72°C for 10 min, with a final hold at 4°C in a DNA thermal cycler (Mastercycler Gradiant, Eppendorf, Germany). The diarrheagenic strain of *B. cereus* (ATCC 14579) was used as a positive control and the sterile water was used as a negative control. PCR products were analyzed in 1.5% (wv^−1^) TAE agarose gels and all PCR experiments were performed twice for each strain.

### 2.4. Statistical Analysis

Statistical analysis was performed using SPSS/18.0 software for significant relationship between hot and cold seasons for occurrence of bacteria in water. Chi-square test was performed and differences were considered significant at  *P* value < 0.05. 

## 3. Results and Discussion

The presence of *B. cereus* in typical colonies has been confirmed by the PCR techniques. In this study, 84 of 200 samples (42%) were found to be contaminated with *B. cereus* between 3 × 10^1^ and 9.3 × 10^1^ (Table 2). Statistical analysis showed significant differences (*P* < 0.05) for the presence and spore ranges of *B. cereus* between baby food with rice and milk based and baby food with wheat, banana, and milk based which was in agreement with the results of Reyes et al. [20]. Reyes et al. [20] showed that 35 out of 56 baby foods with rice and milk based (62.5%) were contaminated with *B. cereus* with a range of 3 to over than 1000 spores per gram sample. Despite the similar results of our study with the study of Reyes et al. [20], the high differences in percentage and range of contamination may be due to the different formulas of the samples, seasons of sample production, methods of sampling, and methods of testing.

The higher contamination of infant foods with cereal based including wheat and rice is maybe due to the higher presence of *B. cereus *in cereal [21]. Various studies showed the high percentages (40%–100%) and ranges (10 to 1000 spores per gram sample) of *B. cereus* in rice [22]. Other studies showed that the *B. cereus* is a flora of rice [23]. In addition, the bacterium has been isolated from another type of cereals (prevalence rate of 56% and contamination range of 10^2^ to 10^5^ spores per gram sample) [24]. It seems that infant food additives have the high role in contamination of baby foods.

Totally, 42% of samples were found to be contaminated with *B. cereus*. Despite the high percentage of *B. cereus* in baby foods in this study, the numbers of spore per gram of each sample were very low and in all samples were lower than 100 spores per gram. The results of Becker et al. [9] were in agreement with our study. They reported that 54% of infant foods were contaminated with *B. cereus* between 3 and 600 spores per gram samples.

Our results showed that *entFM, nheC, bheA, hblC,* and *nheB* with the frequencies of 61.90%, 51.19%, 44.04%, 34.52%, and 33.33% were the highest enterotoxigenic virulence genes isolated from infant foods, respectively (Table 3). This result is in agreement with others [21, 25, 26]. Hansen and Hendriksen [27] showed that 52% of *B. cereus* strains which were isolated from foods had *hblA* genes which were higher than our results. The presence of enterotoxigenic virulence genes in baby food with rice and milk based was the most common while their presence in baby food with wheat, banana, and milk based was rare. Statistical analysis showed significant differences (*P* < 0.05) for the presence of enterotoxigenic virulence genes between baby food with rice and milk based and baby food with wheat, banana, and milk based. Besides, statistical analyses were significant (*P* < 0.05) between the presence of *entFM* with *hblA, hblB,* and *bceT* enterotoxigenic virulence genes in all baby food samples.

In the present study, 14 various patterns have been obtained for detection of enterotoxigenic genes of *B. cereus* in Iranian infant foods (Table 4). These patterns showed that 6.7% of *B. cereus* strains had all studied enterotoxigenic genes while 25.5% of *B. cereus* strains had all studied enterotoxigenic genes except *bceT* gene. This finding is similar to the report of Samapundo et al. [3]. Samapundo et al. [3] showed that 16 various profiles for the presence of *nhe* and *hble * enterotoxigenic genes were obtained from a total of 324 *B. cereus* strains which were isolated from food products in Belgium. They showed that 52.5% of strains had all *hblA, hblB, hblC*, *nheA, nhrB,* and *nheC* enterotoxigenic genes which were in harmony with our results.

The results of the present study showed that despite the high presence of  *B. cereus* in infant food samples, the numbers of spore in gram sample were low. Therefore, more studies with higher numbers of samples are needed to opine about the baby food contamination with *B. cereus*. It seems that some food safety and quality standards (good agricultural practices (GAPs), good manufacturing practices (GMPs), and the hazard analysis and critical control point (HACCP)) systems need to be applied and performed in most of the Iranian infant food units to control growth of *B. cereus* and production of its enterotoxigenic virulence genes during harvesting, distribution, and storage periods. In addition, the hygienic quality of raw materials and even additives are important in reducing the microbial load of infant foods. Also, disinfection of room air, fridge halls, and handling systems is essential. The moisture content of infant foods should be monitored, and storage and transport should be modified according to what has been observed in the models. We suggested that many studies should have been performed on different Iranian foods to study the presence of* B. cereus* and its virulence factors.

## Figures and Tables

**Table 1:** The primer sequences and amplicon sizes used in PCR analysis.

Gene	Primer sequences (50–30) F: forward; R: reverse	Amplicon size (bp)	Reference
*bceT *	F-TTACATTACCAGGACGTGCTT R-TGTTTGTGATTGTAATTCAGG	428	[15]
*entFM *	F-ATGAAAAAAGTAATTTGCAGG R-TTAGTATGCTTTTGTGTAACC	1269	[16]
*hblC *	F-GATACTAATGTGGCAACTGC R-TTGAGACTGCTGTCTAGTTG	740	[17]
*nheA *	F-TACGCTAAGGAGGGGCA R-GTTTTTATTGCTTCATCGGCT	499	[18]
*nheB *	F-CTATCAGCACTTATGGCAG R-ACTCCTAGCGGTGTTCC	769	[18]
*nheC *	F-CGGTAGTGATTGCTGGG R-CAGCATTCGTACTTGCCAA	581	[19]
*hblA *	F-AAGCAATGGAATACAATGGG R-AGAATCTAAATCATGCCACTGC	1154	[17]
*hblB *	F-AAGCAATGGAATACAATGGG R-AATATGTCCCAGTACACCCG	2684	[17]

**Table 2:** Occurrence and distribution of *B. cereus* in various baby food samples.

Samples	Number of samples	*B. cereus* positive (%)	*B. cereus* (spore in gram)	
			Average	Range
Baby food with rice and milk based	50	31 (62)	2.09 × 10^1^	3–9.3 × 10^1^
Baby food with wheat and milk based	50	25 (50)	1.77 × 10^1^	3–9.3 × 10^1^
Baby food with wheat, honey, and milk based	50	18 (36)	2.51 × 10^1^	3–7.5 × 10^1^
Baby food with wheat, banana, and milk based	50	10 (20)	1.61 × 10^1^	2.8–7 × 10^1^

Total	200	84 (42)	1.69 × 10^1^	3–9.3 × 10^1^

**Table 3:** Pattern of enterotoxigenic genes of *B. cereus* isolated from various baby food samples in Isfahan, Iran.

Pattern	Hemolytic enterotoxins			Non-hemolytic enterotoxins			*entFM *	*bceT *
	*hblA *	*hblB *	*hblC *	*nheA *	*nheB *	*nheC *		
1	+	+	+	+	+	+	+	+
2	+	+	+	+	+	+	+	−
3	−	−	+	+	+	+	+	−
4	−	−	−	+	+	+	+	−
5	−	+	+	+	+	+	+	−
6	+	+	+	+	−	+	+	+
7	−	+	+	+	+	+	+	+
8	−	−	+	+	−	+	+	−
9	−	−	−	+	−	+	+	−
10	−	+	+	−	−	−	+	+
11	−	+	+	−	−	−	+	−
12	−	+	−	+	−	−	+	+
13	−	−	−	−	+	−	−	+
14	−	−	−	−	−	−	−	+

**Table 4:** Distribution of various enterotoxigenic virulence genes of *B. cereus* isolated from baby foods in Isfahan, Iran.

Samples	*B. cereus* positive	Enterotoxigenic virulence genes (%)							
		*hb* *lA*	*hb* *lB*	*hb* *lC*	*nh* *eA*	*nh* *eB*	*nh* *eC*	*en* *tF* *M*	*bc* *eT*
Baby food with rice and milk based	31	6	7	10	13	11	17	25	7
		(19.35)	(22.57)	(32.25)	(41.93)	(35.48)	(54.83)	(80.64)	(22.57)
Baby food with wheat and milk based	25	3	4	8	9	8	11	15	5
		(12)	(16)	(32)	(36)	(32)	(44)	(60)	(20)
Baby food with wheat, honey, and milk based	18	1	3	7	9	6	10	7	3
		(5.55)	(16.66)	(38.88)	(50)	(33.33)	(55.55)	(38.88)	(16.66)
Baby food with wheat, banana, and milk based	10	—	1	4	6	4	5	5	1
			(10)	(40)	(60)	(40)	(50)	(50)	(10)

Total	84	11	15	29	37	28	43	52	16
		(13.09)	(17.85)	(34.52)	(44.04)	(33.33)	(51.19)	(61.90)	(19.04)
